# Burden and Predictors of Anemia Among Pregnant and Lactating Females in a Rural Area in India With a High Tribal Population

**DOI:** 10.7759/cureus.67868

**Published:** 2024-08-26

**Authors:** Aishwarya Bhushan, Vidya Sagar, Anit Kujur

**Affiliations:** 1 Community Medicine, Rajendra Institute of Medical Sciences, Ranchi, IND; 2 Preventive Medicine, Rajendra Institute of Medical Sciences, Ranchi, IND

**Keywords:** tribal, rural, lactating, pregnant, predictors, prevalence

## Abstract

Introduction

Nutritional anemia is a silent emergency particularly rampant in developing countries, especially among women of reproductive age group. This study was done with the objective to determine the prevalence and predictors of anemia among pregnant and lactating females in the Ormanjhi block of Ranchi district, Jharkhand.

Methodology

A community-based cross-sectional study was done on 388 pregnant and lactating females from July 2022 to June 2024 using a multi-stage cluster sampling technique. A pre-designed, pre-tested, semi-structured, interviewer-administered questionnaire containing different sections namely socio-demographic details, dietary history, menstrual history, obstetric history, antenatal history, medical history, behavioral history, and personal history was used. House-to-house visits were done for the collection of data. To estimate the prevalence, hemoglobin levels were analyzed using a digital hemoglobinometer.

Results

The overall prevalence of anemia among pregnant and lactating females was found to be 361 (93%) among 388 participants. Prevalence among pregnant females was 295 (92.76%) out of 318, and among lactating females, it was 66(94.28%) out of 70. The prevalence of anemia in the first trimester was 80 (80.45%) out of 87, 112 (94.91%) in the second trimester among 118, and 103 (91.15%) among 113 females in the third trimester.

Conclusion

There are concerns about stagnancy in the prevalence of anemia in pregnancy despite strong political commitments. A baseline data is generated from this study giving a clear picture of the exact prevalence and the predictors of anemia among pregnant and lactating females. This would help the policymakers to make warranted modifications imperative to improve the nutritional status of pregnant and lactating women and hence the children.

## Introduction

Nutritional anemia is a silent emergency particularly rampant in developing countries [1] especially among women of reproductive age group (WRA) affecting nearly two-thirds of pregnant and about half of the non-pregnant females [2]. As per National Family Health Survey (NFHS-5) data (2019-21), the prevalence of anemia is 52% and 57% respectively among pregnant and lactating females. There are 65 districts where anemia is prevalent in >40% of pregnant women [3]. As per global targets 2025, to improve maternal, infant, and young child nutrition, the World Health Organization aims to achieve a 50% reduction in anemia in WRA from a baseline 2012 value of 29% to 15% in 2025, as one of the six global targets [4]. India belongs to high anemia prevalent areas where ≥40% of menstruating adult women and adolescent girls are anemic. India ranked 170 amongst the 180 countries for anemia among females during the Global Nutrition Survey, 2016 [5]. Anemia is a major public health challenge that requires to be addressed from numerous diverse perspectives and through multiple coordinated efforts and actions including government sectors, non-governmental organizations, and United Nations agencies as well as the private sector. Each of these sectors should play a specific and complementary role to collectively achieve anemia reduction and improve health and well-being of not only the beneficiary but of the entire community. National Health Policy of India (2017) [6], in addition to the National Nutrition Strategy (2017) [7], recognizes and addresses anemia as a significantly harmful consequence that could be potentially detrimental to maternal and child survival and also to the productivity of the nation in the long run. There is an urgent need to intensify the existing efforts to address all the possible causes of anemia and thus accelerate the decline in the prevalence of anemia amongst all the included age groups in a mission mode using a multi-pronged strategy [8]. The Indian Government also projects its commitment toward the World Health Assembly target of achieving a 50 percent reduction in anemia among women of reproductive age by 2025 through its Prime Minister Overarching Scheme for Holistic Nutrition (POSHAN) Abhiyaan [9], which is working with a target to reduce the prevalence of anemia among its three beneficiary groups by three percentage points each year. This study was done with the objective to determine the prevalence of anemia among pregnant and lactating females in the Ormanjhi block of Ranchi district, Jharkhand along with their socio-demographic profile and the factors associated with anemia.

## Materials and methods

A community-based cross-sectional study including both descriptive and analytical components was done, including pregnant and lactating females above 18 years of age, residing in various villages of six selected subcenters of Ormanjhi block of Ranchi district in Jharkhand for a 24-month duration starting from July 2022 to June 2024.

The average prevalence of anemia among pregnant and lactating females is 55% [10], with 95% confidence interval (CI) and allowable error/precision of 7%. Considering the design effect of two, a total of 388 subjects were included in the study. Pregnant and lactating females who were willing to participate in the study and gave their written informed consent were included in the study. Individuals diagnosed with serious illness, intellectually challenged, or unable to comprehend the questions asked due to any disability such as hearing impairment were eventually excluded.

Multi-stage cluster sampling was done to achieve this sample size. In the first stage, Ormanjhi Block was selected; then a list of all subcenters was obtained from the Community Health Centre and a list of Anganwadi centers was obtained from the Child Development Project Office of the block. In the second stage, six sub-centers were selected out of 26 sub-centers through a simple random sampling method. In the third stage, a total of 30 villages which come under the six subcenters were selected. Each village was considered as a cluster. Line-listing of all the beneficiaries was obtained from the respective Anganwadi Centre and 13 subjects from each village were included till the sample size of 388 was attained.

A pre-designed, pre-tested, semi-structured, interviewer-administered questionnaire containing different sections namely socio-demographic details, dietary history, menstrual history, obstetric history, antenatal history, medical history, behavioral history, and personal history was used. House-to-house visits were done for the collection of data. If a subject could not be approached for two consecutive visits, we eventually moved to the next individual on the list. Mother and Child Protection (MCP) cards of the beneficiaries were also assessed to confirm the information provided by them. To estimate the prevalence, hemoglobin levels were analyzed using a digital hemoglobinometer (Wrig Nanosystems Pvt. Ltd. Solan, Himachal Pradesh, India).

Data obtained was entered in a template generated in Microsoft Excel 2010 (Microsoft Corporation, Redmond, USA). After data cleaning and coding, data analysis was done using IBM SPSS Statistics for Windows, Version 20 (Released 2011; IBM Corp., Armonk, New York, United States). Qualitative data were expressed in frequency and percentages and quantitative data in mean and standard deviations. Descriptive statistics were studied and frequency tables were generated. Chi-square tests (X2) with appropriate variations were used to assess the significance of associations between the non-parametric variables and the Kruskal-Wallis H test for parametric variables. Based upon the degree of freedom, Cramer’s V or Odd’s ratio (OR) was used as the measure of effect size. A P-value <0.05 was considered to be statistically significant.

Data collection was started after the ethical approval of the Institutional Ethics Committee (IEC), Rajendra Institute of Medical Sciences (RIMS), Ranchi. (Memo no. 13/ IEC/RIMS dated 2/2/23). Written informed consent was obtained from the participants prior to the questionnaire administration.

## Results

At the outset, 399 pregnant and lactating females residing in different villages of six subcenters of Ormanjhi block were approached for interviews. Out of which, six refused to participate, three were chronically ill, and two intellectually challenged females were found. So, they were excluded from the study.

**Figure 1:**
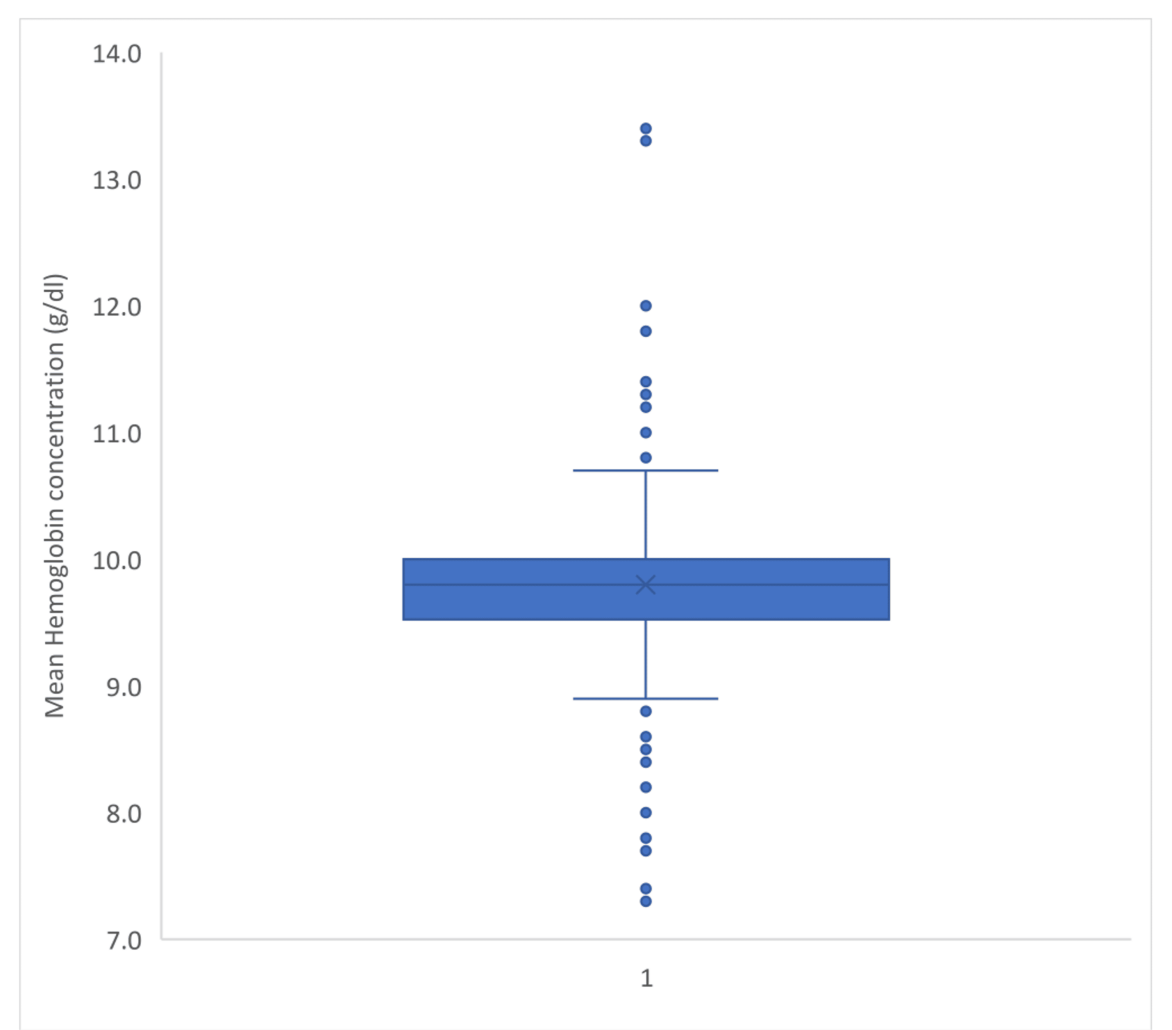
Box and whisker plot showing the hemoglobin level of the study subjects (n=388).

The overall prevalence of anemia among pregnant and lactating females (n=388) was found to be 361 (93%) with 95% CI ranging from 90.4% to 95.6% (Figure 1).

**Figure 2:**
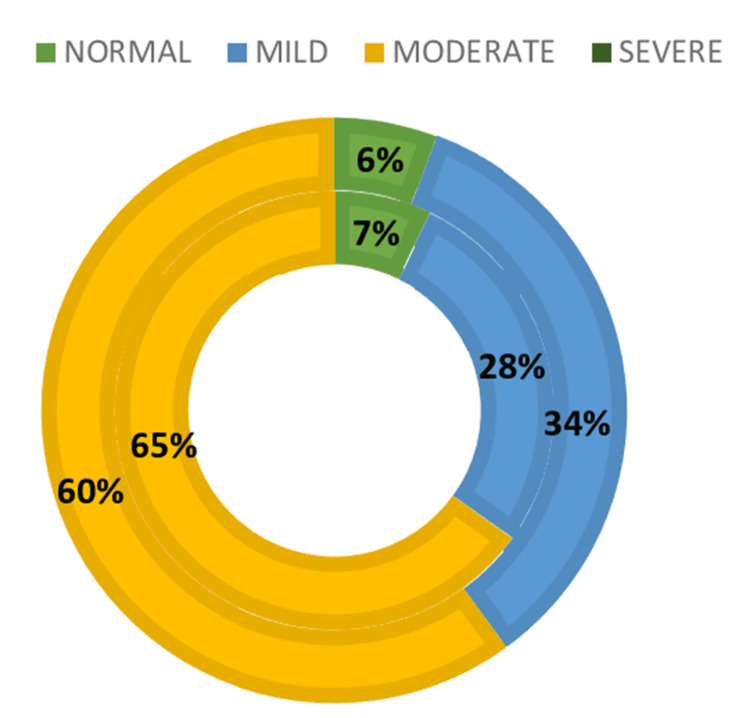
Donut chart showing distribution of subjects according to the severity of anemia (n=388).

The prevalence of anemia among pregnant females (n=318) was found to be 295 (92.76%) with a 95% CI ranging from 90.12% to 95.4% and among lactating females (n=70), it was 66 (94.28%) with a 95% CI ranging from 91.92% to 96.64% (Figure 2).

**Figure 3:**
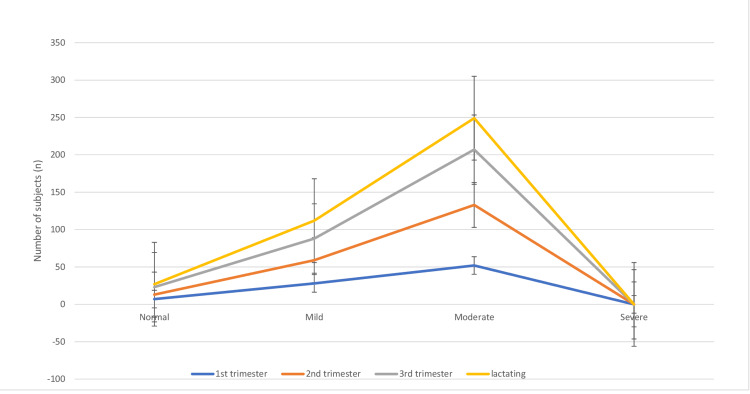
Stacked line chart showing trimester-wise distribution of subjects (n=388).

The prevalence of anemia in the first trimester (n=87) was 80 (80.45%) with 95% CI ranging from 71.85% to 89%. In the second trimester out of 118, the prevalence of anemia was found to be 112 (94.91%) with 95% CI ranging from 90.9% to 98.91%, and 103 subjects (91.15%) out of 113 study subjects were found to be anemic with 95% CI ranging from 85.65% to 96.55% in the third trimester (Figure 3).

Socio-demographic profile

Three hundred and twelve (80.4%) subjects were in the age group of 21 to 34 years (Table 1). The minimum age was 18 years and the maximum age was found to be 40 years. The overall mean age of the study population was 23.76 + 3.53 years with 95% CI (23.40 -24.11). The majority were Hindus 183(47.2%). One hundred and fifty-two subjects (39.4%) were tribal and 235 (60.6%) were non-tribal. The highest percentage of study subjects, 118 (30.4%) had attained education till intermediate, and 260 (67%) were residing in joint families. The majority i.e. 218 (56.2%) of the study subjects were housewives. One hundred and fifty-two subjects (39.2%) belonged to lower middle socio-economic class. Religion and ethnicity were found to be significant predictors of anemia during pregnancy and lactation.

**Table 1:** Socio-demographic profile of study subjects (n=388).

Variables	Category	Frequency (n=388)	Percentage
Age(years)	up to 20 years	73	18.8
	21 to 34 years	312	80.4
	35 years or more	03	0.8
Religion	Hindu	183	47.2
	Muslim	109	28.1
	Christian	3	0.8
	Sarna	93	24.0
Ethnicity	Tribal	153	39.4
	Non-Tribal	235	60.6
Education	Illiterate	16	4.1
	Literate Without Any Formal Education	2	.5
	Primary	12	3.1
	Middle	70	18.0
	Matriculation	99	25.5
	Intermediate	118	30.4
	Graduation	52	13.4
	Post Graduation and Higher	19	4.9
Family Type	Nuclear	119	30.7
	Joint	260	67.0
	Extended	9	2.3
Occupation	Student	14	3.6
	Housewife	218	56.2
	Agriculture	125	32.2
	Daily Wage Laborer	2	.5
	Government Job	1	.3
	Private Job	11	2.8
	Business	17	4.4
Socio-Economic Class	Upper	12	3.1
	Upper Middle	38	9.8
	Middle	76	19.6
	Lower Middle	152	39.2
	Lower	110	28.4

Dietary habits

Three hundred and thirty-nine (87.4%) of the participants were non-vegetarians and 284 (73.2%) of them were primarily rice eaters. Two hundred and sixteen (55.7%) of the pregnant females included jaggery in their diet. Three hundred and thirty participants (85.1%) never ate millets, 171 (44.1%) ate green leafy vegetables (GLV) daily, 6 (1.5%) ate soybean daily, 279 (71.9%) of them consumed it 1 to 3 times in a week, and 102 (26.3%) never ate soybean. Majority i.e. 179 (46.1%) among them never ate iron-rich tubers. Only 180 (46.4%) among them consumed iron-rich dry fruits, 257 (66.2%) of them included iron-rich fruits in their diet, 228 (58.8%) never ate citrus fruits, and majority 225 (58%) among them consumed tea/coffee once or twice a day. Consumption of millets, soybean and green leafy vegetables was found to be significantly associated with anemia (Table 2).

**Table 2:** Predictors of anemia among the subjects (n=361). * Statistically Significant OR: Odds ratio

Variable		Anemic N (%)	Measure of association	P-value	Effect size
Religion	Hindu	174 (48.19)	Stuart Maxwell X^2^= 13.361	0.038*	Cramer’s V= 0.08
	Muslim	96 (26.59)			
	Christian	3 (0.83)			
	Sarna	88 (24.37)			
Ethnicity	Tribal	146 (40.44)	Stuart Maxwell X^2^= 6.901	0.032*	Cramer’s V= 0.09
	Non-tribal	215 (59.55)			
Millet consumption	Yes	57 (15.78)	Fischer’s exact X^2^= 10.036	0.040*	OR= 0.2
	No	304 (84.21)			
Soybean consumption	Yes	268 (74.23)	X^2^= 20.349 (Yates corrected)	0.002*	OR= 0.69
	No	93 (25.76)			
Green leafy vegetable consumption	Yes	358 (99.16)	Fischer’s exact X^2^= 19.306	0.004*	OR=0.1
	No	3 (0.83)			
Comorbidity	Yes	360 (99.72)	Fischer’s exact X^2^= 15.870	0.003*	OR= 0.07
	No	1 (0.27)			

Menstrual and obstetric factors

The mean age at menarche was found to be 12.82 + 1.46. The minimum age was 9 years and the maximum age was 20. The mean menstrual cycle duration of the study subjects was found to be 30.10 + 3.83 days with lowest being 20 days and highest being 60. The mean duration of the menstrual phase was found to be 4.11 + 1.43 days with minimum being 2 and maximum being eight days. Mean gravida of the study population was found to be 2 + 1.05 with minimum being 1 and maximum was 6. Mean parity of the study population was found to be 0.88 + 0.91 with minimum being 0 and maximum was 6. Two-hundred and ninety-eight participants (76.8%) among them never had a history of stillbirth or abortion. These factors were not found to be statistically significant with P-value > 0.05.

Antenatal care (ANC) practices

The majority of them i.e. 108 (27.83%) had only two ANC checkups during pregnancy. Two hundred and nine subjects (53.9%) among them started their iron and folic acid (IFA) supplementation in the second trimester. The majority of them i.e. 64 (25.39%) consumed it for six months only, and 342 (88.14%) pregnant females never ate folic acid in the pregnancy. Only 95 (24.48%) pregnant females consumed the deworming tablets. These factors were not found to be statistically significant (P value > 0.05).

Medical history

Out of the 388 participants, 4 (1.03%) were suffering from hemorrhoids, 2 (0.51%) from menorrhagia and 1(0.25%) from worm infestation. One (0.25%) was suffering from hypertension and 1 (0.25%) from hypothyroidism. The association between presence of comorbidity and anemia was found to be statistically significant with a P-value of 0.003 (Table 2).

Behavioral and personal factors

Three hundred and eighty-six (99.5%) subjects out of 388 practiced handwashing before eating, 379 (97.7%) practiced washing of fruits and vegetables before consuming them raw, 131 (33.8%) used motor to draw their drinking water, followed by 106 (27.3%) who used handpump and 81 (20.9%) who consumed well water, 245 (63.1%) among them practiced purification of water before consumption, 386 (99.5%) subjects practiced handwashing with soap after using washroom, and 262 (67.5%) did not take bath daily. These factors were not found to be statistically significant (P-value > 0.05).

## Discussion

Anemia continues to be a public health exigency despite innumerable programmatic interventions. In our study, the prevalence of anemia among pregnant females was found to be 295(92.76%) out of 318 with 95% CI of 90.12% - 95.4%, and among 70 lactating females, it was 66(94.28%) having 95% Confidence interval of 91.92% - 96.64%. This is similar to the findings reported in the findings reported in studies done by Mangla et al. [10] in 2016, Sinha et al. [11] in 2021, and Kotwal et al. [12] in 2023 who found the prevalence of anemia among pregnant females to be 98%, 90%, and 97.21% respectively. Findings in our study are much higher than the prevalence reported in NFHS-5 which states that the prevalence of anemia among pregnant females in India is 52.2% and among lactating females is 57%. As per NFHS-5, the prevalence of anemia in Jharkhand was found to be 56.8% and 65.3% respectively [13]. Contradictory findings were reported in studies done by Liwey et al. [14] who found 28.3% of lactating females to be anemic. Siddiqui et al. [15] in their study found 59% of pregnant females and 63% of lactating females to be anemic. Kishore et al. [16] and Kamble et al. [17] found the prevalence of anemia among pregnant females to be 33.5% and 59% respectively. None of these studies were done in a tribal-predominant state like Jharkhand. This study depicts the ground reality as the regional communities have their own dietary patterns, endemic infectious diseases, healthcare access, socio-economic disparities, and various other local factors that play an important role.

A statistically significant association was found between religion of the participants and the prevalence of anemia (P-value = 0.038), Cramer’s V=0.08. It is similar to findings reported by Saha et al. [18] (p=0.041). Religious dietary restrictions, fasting practices, and traditional remedies vary from religion to religion thus making it a significant predictor.

The association between ethnicity and anemia was found to be statistically significant (P value = 0.032), Cramer’s V=0.09. It is in compliance with the findings reported by Rahman et al. [19] in their study. This might be because tribal people have their own traditional dietary habits including the food availability and preferences. The existent economic and educational disparities & specific cultural and health related beliefs are important factors.

The association between anemia and millet consumption was found to be statistically significant, OR=0.2 (P-value = 0.040). The same findings were reported in a study done by Sharma et al. [20] (P-value 0.04). The association between anemia and GLV consumption was found to be statistically significant, OR=0.1 (P-value = 0.004). A similar finding was reported in a study done by Grover et al. [21] (P-value 0.001). Soybean consumption was found to be a statistically significant predictor of anemia during pregnancy, OR=0.69 (P-value = 0.002); similar findings were reported in a study done by Lowe et al. [22] (P-value 0.03). High nutrient content, lower phytic acid levels, and the bioavailability of the form of iron found in them are important factors.

The association between the presence of comorbidity and anemia was found to be statistically significant, OR=0.07 (P-value = 0.003). Similar findings were reported in a study done by Pradhan et al. [23]. This finding could possibly be due to increased hemolysis due to chronic strain on blood vessels in case of hypertension and reduced erythropoiesis in hypothyroidism or due to medication side effects.

Limitations of the study were inability to include long-term adherence and outcomes due to time constraints and anthropometric assessment and clinical examination of the subjects would have provided a deeper insight into their nutritional status.

## Conclusions

The alarmingly high prevalence of anemia during pregnancy persists to be a remarkable public health perturb. There exist apprehensions around the torpidity in the prevalence of anemia in pregnancy despite dedicated governmental commitments. The factors contributing to this problem are multifaceted, encompassing individual, socioeconomic, cultural, and organizational or service-related aspects.

Addressing these discrepancies through targeted interventions and enhancing access to healthcare services is imperative. Healthcare systems must be strengthened, medical staff must be adequate, and IFA supplements must be readily available. Boosting awareness, enhancing education, addressing socioeconomic disparities, fostering family support, and building healthcare institutions are all essential components of a comprehensive and multifaceted strategy to combat anemia among pregnant women in India.

## Appendices

Participant ID:                                                                                         MCP card no:

A Study on Coverage of Anemia Mukt Bharat Among Pregnant and Lactating Females in Ormanjhi Block of Ranchi District in Jharkhand

A.  SOCIO-DEMOGRAPHIC DETAILS

A1. Age of the respondent (in completed years): ---------------------

A2. Religion:…………………….. Hindu-1, Muslim-2, Sikh-3, Christian-4, Sarna-5

A3. Ethnicity:…………. Tribal-1, non-tribal-2

A4. Educational status:…………………. Illiterate-1, literate without any formal education-2, up to Primary-3, up to Middle-4, Matric-5, Intermediate-6, Graduate-7, Post graduation -8

A5. Occupation:……………………..Student -1, Housewife-2, Agriculture-3, Daily wage labourer-4, Government service-5, Private job-6, Business-7, Others. Specify-------------------------

A6. Type of family:…………………….. Nuclear-1, Joint-2, Extended-3

A7. Monthly income of the household---------------------------------

A8. Total No. of members in the family--------------------------------

       Per capita income--------------------------

       Modified BG Prasad Classification 2023-----------------------------

B. DIETARY HISTORY

B1. What is your dietary habit:……………… Vegetarian-1, non-vegetarian-2 (if yes, how frequently in a week---------------)

B2. What is your staple food:………………………….. Rice-1, Wheat-2, Maize-3, Pulses-4, Millets-5

B3. Do you include the following food items in your diet

**Table 3:** Food items included in the diet

Food items	Yes-1 No-2	If yes, how frequently in a week do you consume them
Jaggery		
Bajra/jowar/ragi		
Soyabean		
Green leafy vegetables like spinach, beans, sweet potato, beetroot, pumpkin, cabbage		
Dates		
Raisins		
Fig		
Sitaphal		
Pomegranate		
Amla		
Orange		
Lemon		
Tea/coffee		

C. MENSTRUAL HISTORY

C1. Age at first period?    --------------yrs.

C2. What is the average length of your cycle?        ------------------ days

C3. How long does the bleeding last?        ------------------days

C4. How frequently in a day do you need to change the napkin? -----------------

C5. Last menstrual period ------------------------

D. OBSTETRIC HISTORY

D1. Gravida ---------------

D2. Para ----------------

D3.  Type of pregnancy (last pregnancy in case of lactating):……………….Singleton-1, Do not know-2, Others-3. Specify---------------------D4. Gestational month (if lactating, skip this question0:…………………  1st trimester-1, 2nd trimester-2, 3rd trimester-3

D5. Age of the last child born (if primigravida, then skip this question):………………………. Less than 3 years-1, More than 3 years-2

D6. Birth weight of last child born----------------------(skip this question if primigravida)

D7. Did you ever have any abortion or still birth:………………………Yes-1, No-2

E. ANC HISTORY

E1. No. of ANC check-ups done (in last pregnancy if lactating) --------------------------

E2. Hemoglobin level in last ANC visit for pregnant/at the time of delivery for lactating-------------------------

**Table 4:** Iron and folic acid consumption

			IRON	FOLIC ACID
E3	Do you consume the following tablets	Yes-1 No-2		
E4	In which trimester did you start them	1^st^-1 2^nd^-2 3rd -3		
E5	For how many days have you been taking them			
E6	When do you consume the tablet	Empty stomach-1 With food-2 After food-3		

F. MEDICAL HISTORY

F1. Do you take albendazole/mebendazole tablets:…………………………… Yes-1, No-2 (if no, go to Q. F4)

F2. At what time of the day do you chew them:…………………………..After meal at night -1, Any other time-2

F3. How many times in a year do you take them:………………….. Twice-1, Once-2

F4. Are you suffering from any of these conditions:…………………… Haemorrhoids-1, Heavy menstrual bleeding-2, Gastric ulcer-3, Worm infestation-4, Don’t know-5, Others-6.  Specify--------------------------------None-7

F5. Are you suffering from any comorbid condition for which you require regular medication---------------------------------------------------------

G. BEHAVIORAL HISTORY

G1. Do you consume alcohol :……………………… Yes-1, No-2 (if no, go to Q. G5)

G2. Frequency:………………….. Daily -1 (if yes, go to Q. G3, else skip it), Weekly-2, Occasionally -3

G3. What quantity of alcohol do you consume in a day------------------------------

G4. Since how long have you been taking alcohol----------------------

G5. Have you ever been an alcohol consumer in past:…………………………. Yes-1, No-2

G6. Do you consume tobacco:--------------------------Yes-1, No-2 (if no, go to Q. G10)

G7. In which form-------------------------------------------------

G8. Frequency of consumption------------------------------------

G9. For how long have you been consuming tobacco---------------------------------

G10. Did you ever consume tobacco in past:----------------------------------- Yes-1, No-2

H. PERSONAL HISTORY

H1. Do you practice hand washing before eating food:---------------------------------Yes-1, No-2

H2. Do you wash fruits and vegetables before consuming them raw: ------------------------------------- Yes-1, No-2

H3. What is the source of your drinking water: -------------------------------Supply-1, Motor -2, Handpump -3, Well -4, Surface (river/spring)-5, Others-6. specify-------------------------

H4. Do you use any means to purify the water before consumption: --------------------------- Yes-1   How? --------------------, No-2

H5. Do you wash your hands with soap after using washroom: ---------------------------Yes-1, No-2

H6. Do you take bath daily: -----------------------------Yes-1, No-2
